# Is induction of labor associated with poorer maternal satisfaction on labor analgesia? A retrospective study of deliveries with neuraxial analgesia in Helsinki University Hospital delivery units, Finland, 2022

**DOI:** 10.18332/ejm/209667

**Published:** 2025-10-29

**Authors:** Antti Väänänen, Viktoria Sakova, Karoliina Wares, Sirkku Ahlström, Elina Varjola, Riina Jernman

**Affiliations:** 1 Department of Anaesthesiology and Intensive Care, University of Helsinki and Helsinki University Hospital, Helsinki, Finland; 2 Department of Obstetrics and Gynaecology, University of Helsinki and Helsinki University Hospital, Helsinki, Finland

**Keywords:** epidural analgesia, induction of labor, parturient satisfaction

## Abstract

**INTRODUCTION:**

The association of induction of labor with poor maternal satisfaction on analgesia is retrospectively studied in a cohort of parturients delivering with neuraxial analgesia.

**METHODS:**

Satisfaction on analgesia was measured within 1–2 days postpartum and considered as poor (<8/10), fair to good (8–9/10), or excellent (10/10). The incidence of poor maternal analgesia satisfaction was compared by logistic regression following induced (n=2654) or spontaneous onset (n=5222) labors in parturients who delivered with neuraxial analgesia in Helsinki, Finland area hospitals in 2022. Body mass index (BMI), primiparity, diagnosed fear of childbirth (FOC), other modes of labor analgesia, partogram data, and labor outcome were accounted for as cofactors. The association of cofactors within induced cohort was studied by ordinal regression.

**RESULTS:**

The incidence of poor analgesia satisfaction was 24.5% and 19.5% following induction of labor and spontaneous labor, respectively. Adjustment for cofactors (BMI, primiparity, FOC, additional analgesia, cervical dilatation at the time of neuraxial analgesia, intrapartum cesarean delivery) resulted in an AOR for poor satisfaction of 1.19 (95% CI: 1.06–1.34, p<0.001), following induced labor versus spontaneous onset labor. During induced labor, FOC (AOR=1.25; 95% CI: 1.03–1.52), prior opioid labor analgesia (AOR=1.27; 95% CI: 1.09–1.48), cervical dilatation (cm) at the time of neuraxial analgesia (AOR=1.07; 95% CI: 1.02–1.12) and labor resulting in operative vaginal (AOR=1.30; 95% CI: 1.05–1.60) or cesarean delivery (AOR=1.30; 95% CI: 1.06–1.59) were found to be associated with worsening satisfaction, using ordinal regression.

**CONCLUSIONS:**

Induction of labor is associated with higher risk of poor satisfaction on analgesia, and in particular with neuraxial analgesia. Earlier provision of neuraxial analgesia may help mitigate the risk, particularly when additional risk factors are present.

## INTRODUCTION

An increasing number of deliveries are induced, across countries with established healthcare systems^1,2^. When labor is induced the parturient is often admitted to the delivery hospital even before active labor and she is, therefore, within reach of more potent labor analgesia such as systemic opioids and neuraxial analgesia. A recent Finnish birth registry study showed that induction of labor is associated with an increased use of neuraxial analgesia^3^.

One of the reasons underlying the increased use of neuraxial analgesia may be the reported higher pain ratings in early induced labor compared to labor with a spontaneous onset^4,5^. The higher pain scores may result in more parturients being provided with neuraxial analgesia possibly at an earlier phase of labor. A retrospective study by Place et al.^6^ identified pain or insufficient analgesia as the most prevalent reason for poor birthing experience among parturients who delivered in the Helsinki area hospitals in 2017–2018. Interestingly, the proportion of parturients who reported pain as the main reason was lower (OR=0.6; 95% CI: 0.5–0.8) if labor was induced. It was speculated that parturients with induced labor were possibly offered effective analgesia more actively. Since there were no differences in the neuraxial use rate which exceeded 80% in both induced and spontaneous onset labor, this could indicate earlier provision of neuraxial analgesia during induced labor. Unfortunately, neither of the Finnish retrospective studies could address the actual timing of the provision of analgesia^3,6^.

The timing of the analgesia in terms of progression of labor may be of great importance for the satisfaction on analgesia as shown in a prospective Finnish interview study^7^. The study did identify subjective parturient experience of analgesia provision ‘too late’ as a significant risk factor for poor experience on pain relief, an effect that was even more aggravated in parturients with induced labor and attenuated substantially when epidural analgesia was used^7^. Supporting the beneficial effects of earlier neuraxial analgesia, a study by Tan et al.^8^ showed that earlier neuraxial initiation is associated with better maternal satisfaction on analgesia. This was also shown in a large prospective randomized neuraxial analgesia trial by Wang et al.^9^, although the parturients had spontaneous onset labor. Unfortunately, Tan et al.^8^ did not account for induction of labor as a potential confounder and the mean cervical dilatation was only 3.5 cm, which marks a significantly earlier phase of labor compared to the standard practices in our delivery units.

Since neuraxial analgesia use and inductions of labor are on the rise, it is important to study how induction of labor is associated with the parturient satisfaction on analgesia when neuraxial analgesia is used, timing of which may be an important cofactor in satisfaction. In Finland, antenatal maternity care includes screening for potential fear of childbirth (FOC). Experiencing FOC may result in antenatal consultation visits and be associated with earlier admission to the delivery hospital, particularly if FOC is due to fear of pain^10^.

After the delivery the maternal birthing experience and satisfaction on various aspects of the birth, including overall satisfaction with the labor analgesia, is questioned during a routine structured interview. The interview focuses on subjective experiences of the mother on events and actions taken during the labor rather than absolute pain values. The aim of this post-delivery interview is to identify discontented mothers who are in need of a birth experience discussion within the following few months.

The primary outcome of this retrospective study was to analyze the association of induction of labor on maternal satisfaction on analgesia when neuraxial analgesia is used. We considered potential confounding factors, such as body mass index (BMI), multiparity, pre-existing diagnosis for FOC and cervical dilatation at the time of neuraxial analgesia provision. The secondary outcome was to identify possible confounders associated with poorer satisfaction beyond induction within the subgroup of parturients undergoing induced labor.

## METHODS

### Study design and setting

This study is a retrospective cohort analysis incorporating all parturients that used neuraxial labor analgesia during attempted vaginal delivery in four delivery hospitals (Women’s Hospital, Espoo delivery hospital, Lohja hospital, and Hyvinkää hospital) in the Helsinki area between 1 January and 31 December 2022. In this retrospective cohort study, we compare the incidence of poor maternal satisfaction on analgesia in the parturients whose labor is induced against those parturients whose labor has a spontaneous onset. This retrospective study is based on a dataset that was created for a prior study assessing the success of neuraxial analgesia^11^.

### The parturients

A total of 13707 parturients underwent labor. Of these 4035 (29.4%) were induced. Neuraxial analgesia rate was 81.8% and 68.1% following induction and spontaneous onset of labor, respectively.

**Figure 1:**
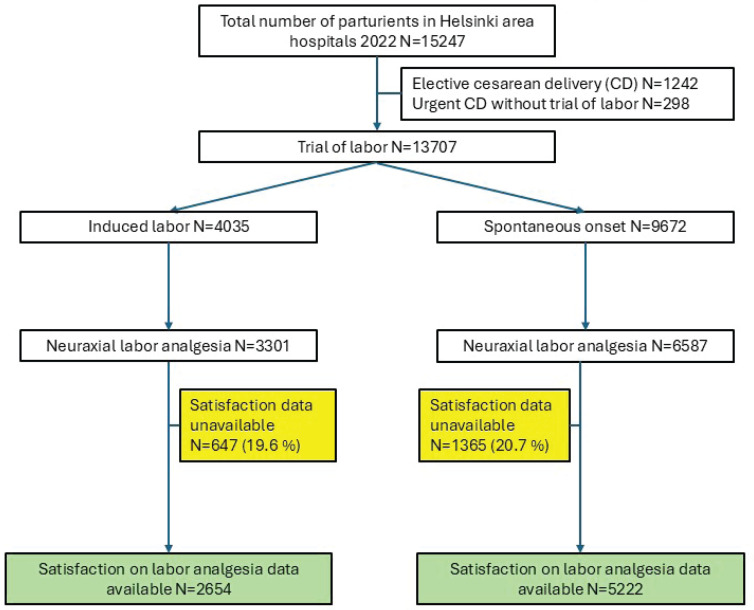
Flowchart of the parturient entry into the study (green) and distribution of the parturients with missing data (yellow) in Helsinki, Finland, area delivery hispitals (2022)

All hospitals in the area use the same obstetric and anesthetic procedures and have full-time anesthetist coverage according to the Finnish legal requirements. Delivery services are offered to all parturients at nominal cost and neuraxial analgesia use does not add to the costs. The flowchart of the parturients is shown in Figure 1.

### Labor analgesia and neuraxial analgesia interventions

The systemic opioid oxycodone may be used in the latent phase of labor given that the fetal heart rate pattern is reassuring, delivery is not expected within the next few hours and the parturient requires analgesia. Typically, oxycodone administration is common in parturients admitted at an early stage of labor due to painful contractions or during induction of labor. The majority (89%) of parturients who receive systemic oxycodone will subsequently receive neuraxial analgesia, typically approximately 2–3 hours later. Oxycodone is not given after the neuraxial analgesia is placed.

Based on the anesthesia procedure notes and medications given, the neuraxial analgesia interventions were classified into:

- Epidural analgesia: A Portex (ICU Medical, San Clemente, CA, USA) 18G closed-tip epidural catheter with multiple lateral holes was used and the initial dose consisted of 20 mg ropivacaine and 0.1 mg fentanyl in a total volume of 20 mL.
- Combined spinal epidural (CSE) analgesia: A Portex CSecure (ICU Medical, San Clemente, CA, USA) needle-through-needle set was used with a 27G pencil point needle. The typical intrathecal dose consisted of 25 ug fentanyl with or without 2.5 mg of bupivacaine in a total volume of 2 mL. Since the intrathecal fentanyl is used primarily in the early phase of labor and the intrathecal fentanyl with bupivacaine in the late phase of labor, these were analyzed separately.
- Single shot spinal analgesia: Consisted typically of 2.5 mg bupivacaine and 25 ug of fentanyl in a volume of 2 mL and used relatively rarely in advanced phase of labor or alternatively used to control the pain at any point of labor to facilitate safe provision of epidural analgesia after the parturient is pain-free.
- Maintenance of epidural analgesia: The analgesia was continued when needed by the midwives using the sited epidural catheters by manual bolus technique. The boluses consisted of 20 mg of ropivacaine and 0.1 mg fentanyl in a total volume of 20 mL given in two portions with at least a five-minute pause between the doses. The use-rate of neuraxial boluses was calculated by considering the initial dose as one dose irrespective of the nature of the intervention. Thereafter, each additional bolus administered by the midwife or anesthetist was calculated as one maintenance bolus and the sum was divided by the number of hours between the intervention and delivery. Any medications given in the operating room in case of operative delivery were omitted from the calculation. The timing of neuraxial analgesia was calculated from the partogram notes and time points before and after the neuraxial analgesia by linear extrapolation and expressed as cervical dilatation state (0–10 cm) at the time of neuraxial analgesia.
- Pudendal block: Ropivacaine (0.2%) 2 mg/mL 10 mL + 10 mL was used for the provision of the pudendal block if additional analgesia was needed for the second phase of labor.

### Collection of data

The satisfaction scores are routinely recorded on the first or the second day after delivery at the maternity ward as a part of the structured pre-discharge interview, where the parturients are asked to mark their birthing experience on a 0–100 mm visual analog scale (0 mm complete dissatisfaction to 100 mm complete satisfaction). Additionally, a separate score is collected for various aspects of the birthing experience, through the questions: ‘Did the delivery go according to your expectations?’, ‘How satisfied were you with the analgesia during labor?’, ‘How well were your wishes and thoughts about your delivery met?’, ‘How well were you included in the decision making during your delivery?’, ‘How was your communication with the midwife at the labor ward?’, ‘How was the communication between the personnel taking care of you at the labor ward?’, ‘How safe did you feel during your delivery?’, ‘Did your support person provide enough support?’, ‘Did you feel being left alone during your delivery?’, and ‘How well did you feel that you were in control during your labor?’. The collection is done by the maternity ward midwives who were not treating the parturient at the labor ward. The numeral rating scale (NRS: 0–10) is used for these sub-parameters with 0 representing complete dissatisfaction and 10 the highest satisfaction.

Parturient demographics (age, height, weight at the time of delivery, gestational age, parity and gravidity, potential prior contacts resulting in diagnosis for FOC) and labor parameters [onset: spontaneous or induced; use of augmentative measures (artificial rupture of membranes or oxytocin infusion); use of labor analgesia by type; partogram notes and time-points; and outcome (spontaneous, instrumental or intrapartum cesarean delivery)] were retrieved from the electronic patient database (Apotti, Epic corporation). All anesthesiological interventions, including the route, dose and timing of neuraxial medications given, were recorded.

### Induction of labor

Induction of labor typically involved cervical ripening with a balloon catheter, prostaglandin or both, followed by rupture of membranes and if needed, oxytocin infusion. Use of any of these measures with the intent of induction of labor constitutes a documented labor induction. Oxytocin infusion is also widely used as augmentation of labor with spontaneous onset and is therefore included in the analysis separately.

### Statistical analysis

Poor maternal satisfaction was defined as maternal satisfaction on analgesia with score <8, while scores 8–9 were considered to represent the intermediate fair-to-good category, and 10 the excellent satisfaction category according to Duale et al.^12^. This corresponds to the scoring used in prior studies on maternal satisfaction on analgesia^7,8^. Statistical analysis was done by SPSS (version 29, IBM).

*Primary and secondary outcomes*

Parturient and labor parameters used for the initial analysis were based on prior studies showing association with either poor satisfaction or function of neuraxial analgesia. A univariate analysis was performed to assess the association of the chosen cofactor candidates with induction of labor. For the primary outcome a univariate binary logistic regression analysis was performed on all initial chosen parameters for the whole 7876 parturient population which included both parturients with induced and spontaneous onset labors with reported satisfaction scores. The analysis was done to predict the maternal analgesia satisfaction score of <8 versus satisfaction score of 8–10, reflecting satisfaction that would be considered ‘poor’ versus ‘better than poor’. Cofactors p<0.05 in the univariate analysis for dissatisfaction were further taken into the multivariate logistic regression analysis step and all parameters that showed a significant association with induction of labor were included as additional cofactors into the model. The model included two parameters describing the use of neuraxial analgesia during labor (duration and number of doses). A derivative of these was formed by calculating the number of doses per hour of neuraxial use and this showed the lowest Akaike information criterion (AIC) value for the overall model, to predict poor maternal satisfaction. No other fitting of the model was done. For the secondary outcome the analysis was performed within the induction and spontaneous onset groups separately, to assess if the relevant cofactors are associate further with poorer satisfaction. An ordinal regression model based on classification of maternal satisfaction as poor (<8), fair to good (8–9), or excellent (10) was used. Potential multicollinearity between cofounders was controlled for by variance inflation factor (VIF) method and the acceptance level was set at VIF <5.

*Handling of missing data*

Adequate partogram and satisfaction data were available from 7876 (80.1%) parturients. The distribution of missing satisfaction data did not differ between induction and spontaneous onset of labor groups (p=0.19). The relatively high number of parturients with no recorded satisfaction score on analgesia could introduce selection bias into the study population and ultimately alter the risk factors. The overall birthing experience score, which is known to correlate well with the labor analgesia satisfaction score^7^, was available from 69.3% of the parturients with missing analgesia satisfaction data. The identified risk factors for poor analgesia satisfaction along with the birthing experience score, when available, were used to impute the missing maternal satisfaction scores (20 iterations). Thereafter, the multivariate analysis for the significant contributors for the poor maternal satisfaction was repeated using the complete pooled data set that included the imputed missing maternal satisfaction on analgesia data.

### Ethical considerations

The study was authorized by the institutional review board for research (Department of Obstetrics, Gynecology and Pediatrics, decisions HUS/614/2023, 8 November 2022, and HUS/707/2025, 23 January 2025). The study was deemed exempt for the requirement for a signed informed consent according to the Finnish research law (488/1999 and 552/2019). All parturient information was provided by the hospital IT administration in a pseudonymized form and processed in a secured environment that met the national requirements for the use of patient health data for scientific purposes, which also abolished the need for ethical board approval (Findata regulation 1/2020, 5 October 2020).

## RESULTS

### The association of induction of labor on poor maternal satisfaction on analgesia

The overall incidence of poor maternal satisfaction was 21.2%. Poor satisfaction was defined by an analgesia satisfaction score <8 on the 0–10 scale (from complete dissatisfaction with analgesia to excellent satisfaction). Following induction of labor, the incidence of poor satisfaction was 24.5% compared to 19.5% after spontaneous onset of labor (OR=1.34; 95% CI: 1.20–1.50, p<0.001).

**Figure 2:**
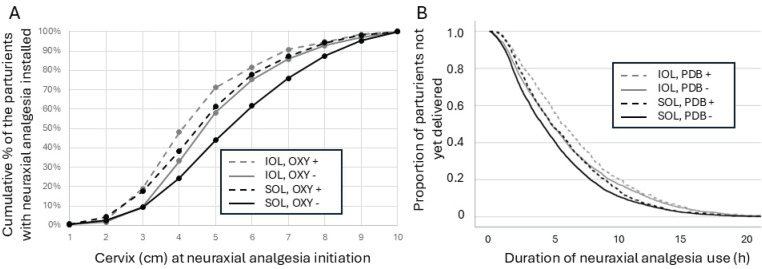
Neuraxial analgesia and prior oxycodone (OXY) and subsequent pudendal block (PDB) analgesia use in induced (IOL) and spontaneous onset labow (SOL) in Helsinki, FInland, area delivery hospitals (2022)

Induction of labor was associated with higher maternal BMI, multiparity, higher incidence for prior diagnosis for FOC, more frequent use of systemic opioids and nitrous oxide in addition to the neuraxial analgesia, initiation of neuraxial analgesia at an earlier cervical dilatation state (Figure 2), longer duration of neuraxial analgesia use, more frequent reciting of the epidural catheter and the labor ending up in intrapartum cesarean delivery more often. The descriptive comparison of the parameters is shown in the Supplementary file Material 1 and univariate analysis for the difference in the parameters in induced labor compared to spontaneous onset labor is shown in the Supplementary file Material 2.

**Figure 3:**
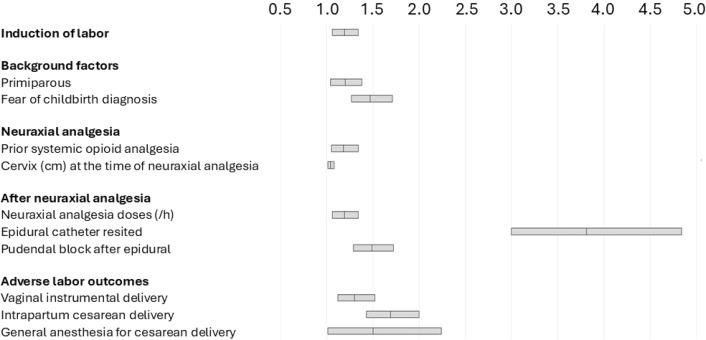
Adjusted odds ratio (95% confidence interval) for poor maternal satisfaction on labor analgesia (vs not-poor) when neuraxial analgesia was used in the Helsinki, FInland, area delivery hospitals in 2022 (N=7876)

To account for the differences in the parturient and labor parameters between parturient populations, a multivariate binary regression model was constructed from the population consisting of both induced and spontaneous onset labors (Table 1). The logistic regression model was statistically significant [χ^2^(16)=326.95, p<0.001]. The model explained approximately 5.8% of the variance in the reported poor satisfaction (Nagelkerke R^2^) and correctly classified 79.1% of the cases. The significant cofactors are shown in Table 1 and Figure 3. In the model adjusted for the other potential cofactors, induction of labor was a significant risk factor for poor maternal satisfaction on analgesia (AOR=1.19; 95% CI: 1.06–1.34, p<0.001).

**Table 1:** Univariate and multivariate logistic regression for poor maternal satisfaction (NRS<8/10 vs 8-10/10) on analgesia in Helsinki area hospitals, Finland, 2022

*Variables*	*Univariate*		*Multivariate*		*Multivariate*	
	*OR (95% CI)*	*p*	*AOR (95% CI)^a^*	*p*	*AOR (95% CI)^a,b^*	*p*
**Total**, n	7876		7876		9833	
**Pre-neuraxial background factors**						
Age (years)	1.00 (0.99–1.01)	0.717				
BMI (kg/m^2^)	1.02 (1.01–1.03)	0.005	1.01 (1.00–1.02)	0.255	1.01 (1.00–1.02)	0.220
Primiparous (vs no)	1.22 (1.08–1.39)	0.002	1.20 (1.04–1.38)	0.012	1.17 (1.02–1.34)	0.025
Prior fear of childbirth dg (vs no)	1.51 (1.30–1.74)	<0.001	1.47 (1.27–1.71)	<0.001	1.43 (1.23–1.66)	<0.001
Induction of labor (vs spontaneous)	1.34 (1.20–1.50)	<0.001	1.19 (1.06–1.34)	0.004	1.20 (1.06–1.36)	0.004
Exogenous oxytocin (vs no)	1.05 (0.94–1.17)	0.420				
**Prior labor analgesia**						
Oxycodone i.m. during labor (vs no)	1.32 (1.18–1.48)	<0.001	1.18 (1.05–1.34)	0.008	1.19 (1.06–1.35)	0.003
**Neuraxial analgesia**						
Cervix at neuraxial initiation (cm)	0.96 (0.94–0.99)	0.009	1.04 (1.01–1.08)	0.023	1.05 (1.01–1.08)	0.012
Resident anesthetist (vs specialist)	1.10 (0.98–1.23)	0.104	1.04 (0.92–1.16)	0.558	1.03 (0.93–1.15)	0.553
**Analgesia type**		<0.001		0.014		<0.001
Epidural analgesia (ref.)	1		1		1	
i.t. fentanyl (CSE)	1.34 (1.19–1.52)	<0.001	1.20 (1.05–1.37)	0.009	1.22 (1.06–1.39)	0.004
i.t. fentanyl and bupivacaine (CSE)	0.82 (0.68–1.00)	0.052	0.91 (0.74–1.11)	0.348	0.90 (0.74–1.11)	0.327
i.t. fentanyl and bupivacaine (single dose)	1.05 (0.75–1.48)	0.780	1.32 (0.89–1.94)	0.165	1.25 (0.85–1.84)	0.261
**Analgesia related post-analgesia events**						
Analgesia duration (h)	1.03 (1.02–1.05)	<0.001				
Neuraxial analgesia use (total doses)	1.06 (1.02–1.11)	0.009				
Neuraxial analgesia doses per hour	1.06 (0.95–1.18)	0.310	1.19 (1.06–1.34)	0.003	1.16 (1.03–1.30)	0.011
Epidural catheter re-sited (vs no)	4.02 (3.18–5.06)	<0.001	3.81 (3.00–4.84)	<0.001	3.74 (2.93–4.77)	<0.001
Epidural blood patch (vs no)	1.80 (0.92–3.49)	0.084				
Pudendal block (vs no)	1.42 (1.24–1.63)	<0.001	1.49 (1.29–1.72)	<0.001	1.49 (1.29–1.71)	<0.001
**Delivery**		<0.001		<0.001		<0.001
Vaginal spontaneous (ref.)	1		1		1	
Vaginal instrumental	1.39 (1.20–1.61)	<0.001	1.30 (1.12–1.52)	<0.001	1.32 (1.13–1.55)	<0.001
Intrapartum cesarean delivery	1.81 (1.57–2.10)	<0.001	1.69 (1.43–2.00)	<0.001	1.68 (1.42–1.99)	<0.001
General anesthesia for cesarean delivery (vs regional anesthesia)	2.34 (1.62–3.37)	<0.001	1.50 (1.01–2.24)	0.046	1.53 (1.04–2.26)	0.031
**Constant**	0.09		0.09			
**Chi-squared (df)**	298.827 (16)	<0.001	360.531 (16)	<0.001		
**Pseudo R^2^ (Nagelkerke)**	0.058		0.055			

AOR: adjusted odds ratio.

a Adjusted for the shown cofactors within column.

b Includes imputed data (pooled from 20 imputations) using the significant cofactors and overall birthing experience score as predictors. BMI: body mass index. CSE: combined spinal epidural analgesia-technique.

### Controlling potential selection bias – missing reported satisfaction values

The satisfaction data were undocumented in 19.6% and 20.7% of the parturients undergoing induction of labor or spontaneous labor, respectively. Estimation of the missing satisfaction levels based on identified risk factors and overall delivery experience score resulted in pooled estimated mean incidence of poor maternal satisfaction in 24.7% of the parturients following induction and 19.6% following spontaneous onset of labor, which align well with the corresponding reported incidences of 24.5% and 19.5%, respectively. The multivariate analysis for the poor satisfaction on analgesia was performed on this pooled dataset which showed that the cofactor estimates fell very near the estimates based on actual measured data (Table 1). Therefore, it was concluded that the missing satisfaction levels unlikely affected significantly the estimation of poor satisfaction associated cofactors and the subsequent analysis for the secondary outcome was performed using the actual measured data.

### The association of cofactors for poorer maternal satisfaction in induced labor

To assess if the potential cofactors for poor satisfaction were associated with worsening of the satisfaction within the induced labor subpopulation, a cumulative odds multivariate ordinal logistic regression model for poorer satisfaction was constructed from the subgroup of parturients who were undergoing induced labor. The model met the requirement for equal odds between the groups as assessed by parallel lines. The model statistically significantly predicted the poorer satisfaction better than the intercept-only model [χ^2^(15)=146.64, p<0.001].

Among the studied parameters higher BMI, the prior diagnosis for FOC, use of oxycodone during labor, administration of neuraxial analgesia at a more advanced cervical dilatation state, use of intrathecal fentanyl rather than epidural analgesia, increasing dosing frequency of the epidural catheter, replacement of the epidural catheter during labor, use of pudendal block for the second phase of labor, and the labor ending up in either instrumental vaginal delivery or intrapartum cesarean delivery were associated with increased risk of poorer maternal satisfaction on analgesia along with the use of general anesthesia during cesarean delivery. The summary of the analysis is shown in Table 2.

**Table 2:** Multivariate ordinal regression for poorer (NRS 10/10 → 8–9/10 → <8/10) maternal satisfaction on analgesia in Helsinki area hospitals, Finland, 2022

*Variables*	*Induction*		*Spontaneous*	
	*AOR (95% CI)^a^*	*p*	*AOR (95% CI)^a^*	*p*
**Total**, n	2654		5222	
**Pre-neuraxial analgesia background factors**				
BMI (kg/m^2^)	1.00 (0.98–1.02)	0.034	1.02 (1.01–1.03)	0.002
Primiparous (vs no)	1.15 (0.97–1.35)	0.112	1.17 (1.03–1.33)	0.017
Prior fear of childbirth dg (vs no)	1.25 (1.03–1.52)	0.022	1.59 (1.36–1.85)	<0.001
**Prior labor analgesia**				
Oxycodone i.m. during labor (vs no)	1.27 (1.09–1.48)	0.002	1.23 (1.09–1.39)	<0.001
**Neuraxial analgesia**				
Cervix at neuraxial initiation (cm)	1.07 (1.02–1.12)	0.009	1.02 (0.99–1.06)	0.143
Resident anesthetist (vs specialist)	1.00 (0.86–1.15)	0.958	1.04 (0.93–1.15)	0.512
**Analgesia type**		0.049		0.225
Epidural analgesia (ref.)	1		1	
i.t. fentanyl (CSE)	1.20 (1.02–1.41)	0.032	1.15 (1.00–1.32)	0.044
i.t. fentanyl and bupivacaine (CSE)	1.00 (0.74–1.36)	0.981	0.97 (0.82–1.15)	0.733
i.t. fentanyl and bupivacaine (single dose)	1.74 (0.95–3.19)	0.072	1.02 (0.73–1.43)	0.891
**Analgesia related post-analgesia events**				
Neuraxial analgesia doses per hour	1.18 (1.01–1.38)	0.034	1.05 (0.94–1.17)	0.422
Epidural catheter re-sited (vs no)	4.18 (2.94–5.94)	<0.001	3.16 (2.37–4.22)	<0.001
Pudendal block (vs no)	1.57 (1.29–1.91)	<0.001	1.30 (1.13–1.50)	<0.001
**Delivery**		0.008		<0.001
Vaginal spontaneous (ref.)	1		1	
Vaginal instrumental	1.30 (1.05–1.60)	0.016	1.42 (1.22–1.64)	<0.001
Intrapartum cesarean delivery	1.30 (1.06–1.59)	0.014	1.78 (1.49–2.14)	<0.001
General anesthesia for cesarean delivery (vs regional anesthesia)	2.45 (1.40–4.28)	0.002	0.96 (0.61–1.51)	0.850
**Chi-squared (df)**	146.643 (15)	<0.001	225.013 (15)	<0.001
**Pseudo R^2^ (Nagelkerke)**	0.061		0.048	

AOR: adjusted odds ratio.

a Adjusted for the shown cofactors within column. BMI: body mass index. CSE: combined spinal epidural analgesia-technique.

### The association of induction of labor on the timing of neuraxial analgesia and other analgesic interventions

The parturients undergoing induced labor received their neuraxial analgesia at an earlier cervical dilatation compared to parturients with spontaneous onset of labor, each cm of cervical progression lowered the likelihood of induced labor (OR=0.86; 95% CI: 0.84–0.88, p<0.001) and showed significantly more frequent use of systemic oxycodone for labor analgesia prior to neuraxial analgesia provision (OR=1.71; 95% CI: 1.55–1.89, p<0.001). Use of systemic opioid was associated with earlier placement of neuraxial analgesia in both induced and spontaneous onset labor (Figure 2A).

Induction of labor was associated with a longer neuraxial analgesia from initiation to delivery (Supplementary file Material 2) but was not associated with differences in pudendal block use rate (p=0.202), which was used in 16.2% of all deliveries. The longer the neuraxial analgesia duration, the higher the likelihood of the parturient receiving also a pudendal block for each hour of neuraxial analgesia (OR=1.04; 95% CI: 1.02–1.05, p<0.001) [χ^2^(1)=25.48, p<0.001]. The association between neuraxial analgesia duration and pudendal block application in induced and spontaneous labor is shown in Figure 2B.

After initiation, neuraxial analgesia was maintained by manual boluses which were used in 83.5% and 75.5% of the parturients in induced and spontaneous onset labor, respectively (OR=1.31; 95% CI: 1.07–1.62, p<0.001). In line with the longer duration of delivery from the initiation of neuraxial analgesia, also the total number of neuraxial analgesia doses was greater in the parturients whose labor was induced. There was no difference between the parturient populations in the total use rate of neuraxial analgesia boluses per hour (Supplementary file Material 2). Increasing the hourly rate of neuraxial boluses was associated with a lower likelihood of the parturient receiving a pudendal block in addition to neuraxial analgesia (OR=0.72; 95% CI: 0.62–0.84, p<0.001). The increasing hourly use of manual epidural boluses over the duration of labor from initiation of analgesia until delivery was associated with greater likelihood of the parturient reporting poor satisfaction on analgesia (AOR=1.19; 95% CI: 1.06–1.14, p<0.003).

## DISCUSSION

Our retrospective analysis shows that despite the use of neuraxial labor analgesia, parturients undergoing induced labor are more likely to rate their labor analgesia experience as poor compared to those parturients whose labor is not induced. The association is not very strong and several parturient and labor specific cofactors further contribute to the poor satisfaction. This likely explains the secondary finding showing that many of the cofactors work beyond induction only and increase the likelihood of poor maternal satisfaction also among those parturients whose labor is induced.

The rating scale for maternal satisfaction on analgesia was based on the meta-analysis by Duale et al.^12^ which suggests that values below 80/100 should be considered to mark ‘poor’ satisfaction on analgesia. This contrasts with the overall birthing experience score where values below 50/100 have been suggested to reflect poor birthing experience in studies where the performance of a simple visual analogue scale value between 0 and 100 has been compared against more complex questionnaires such as the Childbirth Experience Questionnaire (CEQ)^13^. The meta-analysis by Duale et al.^12^ also showed the prevalence of estimated poor (<80/100) satisfaction in 17.8% of the 50531 parturients in material combined from the studies included in the meta-analysis.

The reasons underlying poor satisfaction at an individual parturient level in our study are not known. However, the cofactors showing positive association such as primiparity and fear of childbirth (FOC) may suggest that there are elements where the parturients’ expectations on labor pain and its management are not met. Four main pillars of coping with the labor pain have been identified: preparation, support during labor, sense of control, and being included in the decision-making process^14^. An Icelandic study identified positive attitude to childbirth during pregnancy, use of neuraxial analgesia, lower pain scores during labor and support from the midwife during delivery to be associated with a positive experience with labor pain^15^.

All parturients in our study cohort had neuraxial labor analgesia. The personal preferences of the parturient are requested in a pre-delivery form addressing several aspects of the delivery, including thoughts about labor pain and preferences for analgesic choices. The neuraxial analgesia use rate was 74% and often the primiparous parturients may be hesitant about the neuraxial procedure, which may partly explain the popularity of using milder methods, such as systemic opioids, in the early phase of labor. Additionally, the parturients may have very precise wishes regarding their delivery and analgesia resulting in disappointment should these wishes not materialize. A prior study has shown that parturients who did not plan on using neuraxial analgesia but changed their mind during delivery were less satisfied with their analgesia and such discrepancy between expectations and outcome may even increase the risk of postpartum depression^16,17^. These phenomena may at least partly explain the higher rating of poor satisfaction in primiparous parturients compared to multiparous parturients (Table 1).

The choice of analgesic can be problematic in the early stages of labor. Giving that the systemic opioid is strongly associated with receiving neuraxial analgesia later on during the delivery and since postponing the initiation of neuraxial analgesia is associated with worse satisfaction ratings, it would seem beneficial to replace the early opioid with direct neuraxial analgesia. While this may be sensible in some cases, the practice would prolong the time the parturient spends in the labor room as the neuraxial analgesia requires currently that the parturient is admitted to the labor room and is subject to continuous fetal heart rate monitoring. Thus, the initiation of neuraxial analgesia may be controlled to some extent by hospital resources (availability of the labor rooms) during busy times.

One of the cofactors that challenges the parturients’ resources to cope with labor pain is fear of childbirth (FOC), the incidence of which is on the rise in Finland^18^. The increasing rate of elective cesarean deliveries performed due to FOC has resulted in implementation of a treatment system where the primary healthcare maternity services screen the expecting women for FOC, and if FOC is suspected the woman is referred to a specialized clinic at the maternity hospital. The actual diagnosis for FOC is only set at this clinic after counselling. Various factors may contribute to FOC, such as worries about the safety of the child, fear of loss of self-control, and pain^19^. Parturient with a diagnosed FOC is also more likely to undergo induction of labor (OR=1.42; 95% CI: 1.25–1.62), since some of the inductions are done due to maternal request to control fears for reasons such as fetal macrosomia or not being able to travel to delivery hospital in time. FOC has been shown to be associated with pain catastrophizing tendency, as well as higher anticipated and reported pain values during labor compared to parturients without FOC^20-23^. Therefore, compared to parturients without FOC, adequate pain relief is an even more critical requirement in attempting vaginal delivery and should thus be provided at an early stage.

In addition to FOC, induction of labor is associated with obesity, multiparity, use of exogenous oxytocin, initiation of the labor analgesia with systemic opioids and placement of the neuraxial analgesia at an earlier phase of labor compared to parturients with a spontaneously onset labor (Supplementary file Material 2). Our multivariate analysis (Table 1) suggests that obesity and exogenous oxytocin do not show an association with poor analgesia satisfaction, while multiparity and early neuraxial analgesia are associated with reduction of the poor satisfaction risk. The institutional guidelines promote early insertion of an epidural analgesia for the obese parturients, to facilitate relatively rapid and reliable anesthesia for peripartum cesarean delivery if needed^24^. Thus, obese parturients may be actively offered neuraxial analgesia at an early stage of labor.

The overall ratio of parturients with poor satisfaction on analgesia was 21.2%, which corresponds relatively well with the prior study of Mäkelä et al.^7^ from another university hospital in Finland whose prospective study had 35.5% and 32.4% of the parturients report a satisfaction NRS rating of <8/10 after induced and spontaneous labor, respectively^7^. It is noteworthy that in their study, the sample was taken from parturients that had eventually delivered vaginally and only a portion of the parturients had neuraxial analgesia. The prevalence of poor satisfaction was lower in our study compared to a study from Singapore^8^. The authors showed that 31.8% of the parturients reported satisfaction scores of <8/10 when neuraxial analgesia was used. Meanwhile, many of the risk factors for the poor satisfaction score were similar to those of our study. Regarding neuraxial analgesia, the pain ratings prior to it did not relate to satisfaction, whereas pain ratings afterwards did^8^. In our study, the prior use of systemic opioid for labor analgesia, a surrogate for significant pain prior to neuraxial analgesia, did correlate with poorer satisfaction scores as did the need for additional analgesia procedure, pudendal block. The difference compared to the study by Tan et al.^8^ may be their initiation of neuraxial analgesia at a markedly earlier stage of cervical dilatation compared to our cohort, which could hypothetically also lead to better sacral spread of the analgesia over time.

The findings of our study may have implications for institutional practices and protocols to consider if a parturient should receive neuraxial analgesia earlier – maybe even replacing the systemic opioid. However, on an individual level, the multivariate model may provide relatively little help with decision making as the absolute risk increase for the parturient to report poor satisfaction on analgesia is relatively modest (2.9%) based on the baseline incidence of 19.5% and an AOR=1.19. As shown in Figure 3, multiple factors are associated with the likelihood of reporting the satisfaction as poor and a parturient with induced labor would have her increased risk of dissatisfaction mitigated if the often-used systemic opioid analgesia would be replaced with a direct neuraxial, preferably traditional epidural, analgesia. This approach would be very close to the previously published large, randomized studies comparing early neuraxial analgesia with an approach where the analgesia is initiated with systemic opioid and a neuraxial analgesia at 4–5 cm cervical dilatation^9,25^. Based on these studies, it is safe to conclude that giving the neuraxial analgesia at the time when the systemic opioid is typically administered in our parturient population, no adverse outcomes regarding duration or outcome of labor should be seen^9,25^. Meanwhile, in the prospective randomized study of 12793 parturients by Wang et al.^9^, earlier initiation of epidural analgesia was associated with better maternal satisfaction.

Careful assessment of the functionality of the epidural catheter should be emphasized since the need to replace the epidural catheter was the strongest identified cofactor for poor satisfaction on analgesia (Table 1, Figure 3). There was substantial distribution in the individual hourly consumption of the manual epidural maintenance boluses and induction of labor was associated with longer use time of neuraxial analgesia (Figure 2B). One potential approach that could improve both parturient self-control, satisfaction and help meet the individually required amount of epidural analgetic would be to switch from the manual bolus-technique to parturient controlled pump-driven epidural maintenance system^9,26^.

### Limitations

The main limitation of our study involves its retrospective nature. The quality of the data reflects the accuracy and thoroughness of data stored in the patient records. However, all data used for this study are collected routinely as part of the normal treatment of the parturients and most are mandatory and validated, as the data are relayed to the national Medical Birth Register (Finnish Institute for Health and Welfare). Due to the retrospective nature of this study no confirming assumptions about causality of events can be drawn. For example, it is unlikely that initiation of neuraxial analgesia by intrathecal fentanyl alone and continuation with epidural analgesia thereafter would cause worse satisfaction on analgesia compared to traditional epidural analgesia. The parturients who were chosen to have the intrathecal fentanyl initiation for their neuraxial analgesia were more often primiparous, undergoing induced labor and received their analgesia at an earlier phase of labor as was shown in a prior study comparing the success of the epidural and CSE analgesia in the same database^11^. Another limitation is that the performance of epidural analgesia may be somewhat dependent on the personal practices and attitudes of the midwife caring for the parturient and success of the communication between the parturient and her midwife about the anticipations regarding labor pain and its management. This communication may be severely challenged by the language barrier between the parturient and her caretakers as approximately 25.6% of the parturients that delivered during the study period were non-native Finnish or Swedish speakers. The same reason may explain, to some extent, the level of missing data in the satisfaction scores. Along with different languages, the immigrant parturient population has its own cultural expectations on the management of labor and labor pain, which may be difficult to communicate to the healthcare staff if there is no common language.

Regarding generalizability of our results, socioeconomic factors have not been shown to influence neuraxial labor analgesia use in the publicly funded Finnish healthcare system^27^. Our results, which are based on the cohort of parturients with neuraxial analgesia use, may not therefore be directly generalizable to a system with cost barriers potentially limiting neuraxial analgesia use.

## CONCLUSIONS

Induction of labor is associated with higher likelihood of poor satisfaction on labor analgesia, including when neuraxial analgesia is used. The association is not very strong, possibly owing to earlier onset of neuraxial analgesia during induced labor compared to labor with spontaneous onset. The use of systemic opioid prior to neuraxial analgesia, onset of neuraxial analgesia at a later phase of labor, replacement of the epidural catheter during labor, need for pudendal block in addition to the neuraxial analgesia and ending up in instrumental vaginal delivery or operative delivery, were associated with poorer maternal satisfaction on analgesia also in parturients whose labor was initially induced. The onset of neuraxial analgesia is one of the few analgesia satisfaction-associated parameters that can be easily modified. Our results suggest that in parturients whose labor is induced, early epidural analgesia should be offered, particularly if other known risk factors for poor analgesia satisfaction are present.

## Supplementary Material



## Data Availability

The data supporting this research cannot be made available for privacy or other reasons.
