# Supplementary material for: Is induction of labor associated with poorer maternal satisfaction on labor analgesia? A retrospective study of deliveries with neuraxial analgesia in Helsinki University Hospital delivery units, Finland, 2022
Source: Eur J Midwifery. 2025 Oct 29;9:10.18332/ejm/209667. doi: 10.18332/ejm/209667 (PMC12569814; doi:10.18332/ejm/209667)
Supplement: Supplementary file 1 [file EJM-9-47-s1.pdf]

Supplementary Table 1

Parturient, labor, and labor analgesia information for the 7876 parturients with known satisfaction score in Helsinki, Finland, area delivery hospitals in 2022

Onset of labor	Induced or spontaneous (all parturients in the study)				Induced				Spontaneous				*Induced vs spontaneous*
Satisfaction category	Poor	Fair-Good	Excellent	*All*	Poor	Fair-Good	Excellent	*All*	Poor	Fair-Good	Excellent	*All*	*All*
Satisfaction numeral rating scale value	<8/0-10	8-9/0-10	10/0-10		<8/0-10	8-9/0-10	10/0-10		<8/0-10	8-9/0-10	10/0-10		
N	1670	3065	3141	*7876*	651 (24.5 %)	1059 (39.9 %)	944 (35.6 %)	*2654*	1019 (19.5 %)	2006 (38.4 %)	2197 (42.1 %)	*5222*	*p-value*
**Pre-neuraxial analgesia factors**													
Background factors													
Age (yr)	32.4 (5.0)	32.4 (5.0)	32.6 (5.0)	*32.5 (5.0)*	32.7 (5.0)	32.4 (4.9)	32.4 (4.9)	*32.5 (5.0)*	32.3 (4.8)	32.4 (4.9)	32.6 (4.9)	*32.5 (4.9)*	*0.475*
BMI (kg/m2)	25.7 (5.5)	25.5 (5.2)	25.1 (4.9)	*25.4 (5.2)*	26.6 (6.0)	26.7 (5.9)	26.4 (5.9)	*26.6 (5.9)*	25.1 (5.0)	24.9 (4.6)	24.5 (4.3)	*24.8 (4.6)*	*<0.001*
Primiparous	1290 (77.2 %)	2294 (74.8 %)	2267 (72.2 %)	*5851 (74.3 %)*	477 (73.3 %)	735 (69.4 %)	637 (67.5 %)	*1849 (69.7 %)*	813 (79.8 %)	1559 (77.7 %)	1630 (74.2 %)	*4002 (76.6 %)*	*<0.001*
Prior fear of childbirth dg	297 (17.8 %)	432 (14.1 %)	347 (11.0 %)	*1076 (13.7 %)*	117 (18.0 %)	187 (17.7 %)	134 (14.2 %)	*438 (18.5 %)*	180 (17.7 %)	245 (12.2 %)	213 (9.7 %)	*638 (12.2 %)*	*<0.001*
Exogenous oxytocin	752 (45.0 %)	1390 (45.4 %)	1336 (42.5 %)	*3478 (44.2 %)*	449 (69.0 %)	805 (76.0 %)	708 (75.0 %)	*1962 (73.9 %)*	303 (29.7 %)	585 (29.2 %)	628 (28.6 %)	*1516 (29.0 %)*	*<0.001*
**Prior labor analgesia**													
Oxycodone i.m. during labor	577 (34.6 %)	958 (31.3 %)	812 (25.9 %)	*2347 (29.8 %)*	269 (41.3 %)	414 (39.1 %)	311 (32.9 %)	*994 (37.5 %)*	308 (30.2 %)	544 (27.1 %)	501 (22.8 %)	*1353 (25.9 %)*	*<0.001*
**Neuraxial analgesia**													
Cervix at neuraxial initiation (cm)	5.51 (1.99)	5.61 (1.89)	5.69 (1.87)	*5.62 (1.90)*	5.3 (1.8)	5.3 (1.7)	5.3 (1.7)	*5.3 (1.8)*	5.6 (2.1)	5.8 (1.9)	5.9 (1.9)	*5.8 (2.0)*	*<0.001*
Resident anesthetist	660 (39.5 %)	1157 (37.7 %)	1162 (37.0 %)	*2979 (37.8 %)*	252 (38.8 %)	436 (41.2 %)	362 (38.4 %)	*1050 (39.6 %)*	408 (40.2 %)	721 (36.1 %)	800 (36.6 %)	*1929 (37.1 %)*	*0.027*
Analgesia type													
Epidural analgesia	944 (56.5 %)	1821 (59.4 %)	1937 (61.7 %)	*4702 (59.7 %)*	337 (51.8 %)	568 (53.6 %)	552 (58.5 %)	*1457 (54.9 %)*	607 (59.6 %)	1253 (62.5 %)	1385 (63.0 %)	*3245 (62.1 %)*	*<0.001*
i.t. fentanyl (CSE)	544 (32.6 %)	850 (27.7 %)	763 (24.3 %)	*2157 (27.4 %)*	259 (39.8 %)	401 (37.9 %)	313 (33.2 %)	*973 (36.7 %)*	285 (28.0 %)	449 (22.4 %)	450 (20.5 %)	*1184 (22.7 %)*	*<0.001*
i.t. fentanyl and bupivacaine (CSE)	139 (8.3 %)	319 (10.4 %)	353 (11.2 %)	*811 (10.3 %)*	40 (6.1 %)	74 (7.0 %)	67 (7.1 %)	*181 (6.8 %)*	99 (9.7 %)	245 (12.2 %)	286 (13.0 %)	*630 (12.1 %)*	*<0.001*
i.t. fentanyl and bupivacaine (single dose)	43 (2.6 %)	75 (2.4 %)	88 (2.8 %)	*206 (2.6 %)*	15 (2.3 %)	16 (1.5 %)	12 (1.3 %)	*43 (1.6 %)*	28 (2.7 %)	59 (2.9 %)	76 (3.5 %)	*163 (3.1 %)*	*<0.001*
**Analgesia related post analgesia events**													
Analgesia duration (h)	6.16 (4.74)	5.79 (4.21)	5.32 (3.81)	*5.68 (4.19)*	6.6 (4.8)	6.3 (4.5)	6.0 (4.3)	*6.3 (4.5)*	5.9 (4.7)	5.5 (4.0)	5.0 (3.5)	*5.4 (4.0)*	*<0.001*
Neuraxial analgesia use (total doses)	2.88 (1.32)	2.82 (1.20)	2.76 (1.11)	*2.81 (1.19)*	3.06 (1.28)	2.93 (1.16)	2.87 (1.03)	*2.95 (1.15)*	2.76 (1.33)	2.76 (1.21)	2.71 (1.14)	*2.74 (1.21)*	*<0.001*
Manual maintainance boluses per hour	0.41 (0.59)	0.39 (0.46)	0.41 (0.44)	*0.40 (0.48)*	0.43 (0.54)	0.41 (0.50)	0.41 (0.41)	*0.42 (0.48)*	0.40 (0.62)	0.29 (0.44)	0.41 (0.45)	*0.40 (0.49)*	*0.320*
Epidural catheter resited	152 (9.1 %)	98 (3.2 %)	53 (1.7 %)	*303 (3.8 %)*	71 (10.9 %)	42 (4.0 %)	16 (1.7 %)	*129 (4.9 %)*	81 (7.9 %)	56 (2.8 %)	37 (1.7 %)	*174 (3.3 %)*	*<0.001*
Epidural blood patch	13 (0.8 %)	12 (0.4 %)	15 (0.5 %)	*40 (0.5 %)*	4 (0.6 %)	2 (0.2 %)	4 (0.4 %)	*10 (0.4 %)*	9 (0.9 %)	10 (0.5 %)	11 (0.5 %)	*30 (0.6 %)*	*0.243*
Pudental block	337 (20.2 %)	502 (16.4 %)	435 (13.8 %)	*1274 (16.2 %)*	144 (22.1 %)	179 (16.9 %)	126 (13.3 %)	*449 (16.9 %)*	193 (18.9 %)	323 (16.1 %)	309 (14.1 %)	*825 (15.8 %)*	*0.202*
Delivery													
Vaginal spontaneous	1052 (63.0 %)	2140 (69.8 %)	2390 (76.1 %)	*5582 (70.9 %)*	386 (59.3 %)	690 (65.2 %)	649 (68.8 %)	*1725 (65.0 %)*	666 (65.4 %)	1450 (72.5 %)	1741 (79.2 %)	*3857 (73.9 %)*	*<0.001*
Vaginal instrumental	288 (17.2 %)	503 (16.4 %)	389 (12.4 %)	*1180 (15.0 %)*	107 (16.4 %)	172 (16.2 %)	120 (12.7 %)	*399 (15.0 %)*	181 (17.8 %)	331 (16.5 %)	269 (12.2 %)	*781 (15.0 %)*	*0.927*
Intrapartum cesarean delivery	330 (19.8 %)	422 (13.8 %)	362 (11.5 %)	*1114 (14.1 %)*	158 (24.3 %)	197 (18.6 %)	175 (18.5 %)	*530 (20.0 %)*	172 (16.9 %)	225 (11.2 %)	187 (8.5 %)	*584 (11.2 %)*	*<0.001*
General anesthesia for cesarean delivery	47 (2.8 %)	41 (1.3 %)	35 (1.1 %)	*123 (1.6 %)*	24 (3.7 %)	16 (1.5 %)	10 (1.1 %)	*50 (1.9 %)*	23 (2.3 %)	25 (1.2 %)	25 (1.1 %)	*73 (1.4 %)*	*0.100*
**Birthing experience**													
Experience score (0-100, mean; SD)	68 (25)	82 (21)	88 (23)	*81 (24)*	67 (24)	81 (22)	87 (24)	*80 (25)*	69 (25)	83 (20)	88 (23)	*82 (23)*	
Experience <50/100 (N (%))[a]	254 (16.2 %)	180 (6.3 %)	204 (6.9 %)	*638 (8.7 %)*	97 (16.1 %)	75 (7.6 %)	66 (7.5 %)	*238 (9.6 %)*	157 (16.3 %)	105 (5.6 %)	138 (6.7 %)	*400 (8,2 %)*	

[a] The birthing experience score was available from 2470 and 4902 parturients in the induced and spontaneous groups, respectively.

Supplementary Table 2

Univariate logistic regression for induction of labor (vs spontaneous onset) in Helsinki, Finland, area hospitals in 2022

	OR (95 % CI)	p
N	7876	
**Pre-neuraxial analgesia factors**		
Background factors		
Age (yr)	1.00 (0.99-1.01)	0.950
BMI (kg/m2)	1.07 (1.06-1.08)	<0.001
Primiparous (vs no)	0.70 (0.63-0.78)	<0.001
Prior fear of childbirth dg (vs no)	1.42 (1.25-1.62)	<0.001
Exogenous oxytocin (vs no)	6.93 (6.24-7.70)	<0.001
**Prior labor analgesia**		
Oxycodone i.m. during labor (vs no)	1.71 (1.55-1.89)	<0.001
**Neuraxial analgesia**		
Cervix at neuraxial initiation (cm)	0.86 (0.84-0.88)	<0.001
Resident anesthetist (vs specialist)	1.11 (1.01-1.23)	0.027
Analgesia type		<0.001
Epidural analgesia (reference)	1	<0.001
i.t. fentanyl (CSE)	1.83 (1.65-2.03)	<0.001
i.t. fentanyl and bupivacaine (CSE)	0.64 (0.64-0.76)	<0.001
i.t. fentanyl and bupivacaine (single dose)	0.59 (0.42-0.64)	0.002
**Analgesia related post analgesia events**		
Analgesia duration (h)	1.05 (1.04-1.06)	<0.001
Neuraxial analgesia use (total doses)	1.16 (1.11-1.20)	<0.001
Manual maintenance boluses (/h)	1.07 (0.98-1.18)	0.145
Epidural catheter resited (vs no)	1.48 (1.17-1.87)	<0.001
Epidural blood patch (vs no)	0.66 (0.32-1.34)	0.247
Pudendal block (vs no)	0.92 (0.81-1.05)	0.202
Delivery		<0.001
Vaginal spontaneous (reference)	1	
Vaginal instrumental	1.14 (1.00-1.31)	0.050
Intrapartum cesarean delivery	2.03 (1.781-2.31)	<0.001
Anesthesia for cesarean delivery		
General anesthesia (vs regional)	1.35 (0.94-1.95)	0.101

© 2025 Väänänen A. et al.
